# Gender Predicts Differences in Acute Ischemic Cardioembolic Stroke Profile: Emphasis on Woman-Specific Clinical Data and Early Outcome—The Experience of Sagrat Cor Hospital of Barcelona Stroke Registry

**DOI:** 10.3390/medicina60010101

**Published:** 2024-01-05

**Authors:** Marc Inogés, Adrià Arboix, Luís García-Eroles, María José Sánchez-López

**Affiliations:** 1 Department of Neurology, Hospital Universitari Sagrat Cor, Grupo Quirónsalud, Universitat de Barcelona, 08029 Barcelona, Catalonia, Spain; minogesv@gmail.com (M.I.); lgarciaer@gmail.com (L.G.-E.); 2 Medical Library, Hospital Universitari Sagrat Cor, Grupo Quirónsalud, Universitat de Barcelona, 08029 Barcelona, Catalonia, Spain; biblioteca.hsc@quironsalud.es

**Keywords:** acute cerebrovascular event, cardioembolic stroke, estrogens, gender, ischemic stroke, men, sex differences, stroke data, stroke registry, women

## Abstract

*Background and Objectives*: Acute ischemic cardioembolic stroke (CS) is a clinical condition with a high risk of death, and can lead to dependence, recurrence, and dementia. *Materials and Methods*: In this study, we evaluated gender differences and female-specific clinical data and early outcomes in 602 women diagnosed with CS from a total of 4600 consecutive acute stroke patients in a single-center hospital stroke registry over 24 years. A comparative analysis was performed in women and men in terms of demographics, cerebrovascular risk factors, clinical data, and early outcomes. *Results*: In a multivariate analysis, age, hypertension, valvular heart disease, obesity, and internal capsule location were independent variables associated with CS in women. The overall in-hospital mortality rate was similar, but the group of women had a greater presence of neurological deficits and a higher percentage of severe limitation at hospital discharge. After the multivariate analysis, age, altered consciousness, limb weakness, and neurological, respiratory, gastrointestinal, renal, cardiac and peripheral vascular complications were independent predictors related to early mortality in women. *Conclusions*: Women with CS showed a differential demographic and clinical profile and worse early outcomes than men. Advanced age, impaired consciousness, and medical complications were predictors of stroke severity in women with CS.

## 1. Introduction

Stroke is one of the leading causes of increase in acquired disability, and, in turn, acute cerebrovascular diseases, mostly ischemic in nature, are the second leading cause of death in wealthy countries in both men and women, although deaths have been reduced by 21% [1,2,3,4]. The precise definition of the stroke subtype and underlying mechanism is crucial, as it will determine the most effective care and therapy [5,6,7,8,9,10].

The most common origin of cerebral embolism is one of three mechanisms: blood stasis and thrombus formation in the left heart chambers caused by rheumatic valve disease, myocardial infarction, and sequelae and cardiac rhythm disturbances; the release of material from an abnormal valve surface (e.g., calcified degeneration); and anomalous passage from the venous to the arterial circulation (paradoxical embolism) [11,12,13,14]. Emboli originating in the cardiac cavities are usually large and thus responsible for causing strokes of greater severity, with an added risk of disability and, ultimately, death.

Cardioembolic stroke is the most severe subtype of ischemic stroke, with a high probability of neurological dysfunction at hospital discharge, a non-negligible risk of early embolic recurrence (1–10%) [14,15,16], and, at the same time, the subtype with the highest in-hospital mortality (6–27%) [1,3,10]. Despite all this and accounting for up to a quarter of all cerebral infarctions [10,11], some clinical aspects of the natural course of the disease remain poorly defined.

The percentage of cardioembolic strokes in the Stroke Registry of the Hospital Sagrat Cor de Barcelona was 18% [17], being similar to that reported in the studies of Timsit et al. and Bougousslavsky et al. (19.4% and 16%, respectively) [18,19]; this frequency is higher than that found in the studies by Vázquez et al. and Al-Rajed et al. (14% in both cases) [20,21] and, in turn, lower than the percentages reported by Rothrock et al. and Norrving and Löwenhielm (22% and 30.6%, respectively) [22,23].

Age is a risk factor in the incidence of cardioembolic cerebral infarction. In the oldest group of patients (≥85 years), it was the most frequent ischemic subtype, accounting for 36% of all strokes, whereas in those younger than 65 years, the incidence of cardioembolic infarction accounted for 14.6% [24].

Although there are no absolute criteria for the diagnosis of cardioembolic cerebral infarction, there is consensus in requiring three basic elements: a compatible clinical picture, a recognition of embolic heart disease, and an exclusion of carotid and/or cerebral atherosclerosis or another cause of the infarction [3,10,25].

Clinical features supporting the diagnosis of cardioembolic stroke include sudden onset to maximum deficit (<5 min), present in 47–74% of cases, and a decreased level of consciousness at onset, in 19–31% of cases [26]. A classic cardioembolic presentation includes symptom onset following Valsalva-provoked activity (coughing, bending over, etc.), suggestive of paradoxical embolism facilitated by a transient increase in right atrial pressure and the co-occurrence of cerebral and systemic emboli [10,24].

The most frequent high-risk cardioembolic conditions are atrial fibrillation, recent myocardial infarction, mechanical valve prosthesis, dilated cardiomyopathy, and rheumatic mitral stenosis. Other important sources of cardioembolism are infective endocarditis, marantic endocarditis, and atrial myxoma; and minor sources are patent foramen ovale, atrial septal aneurysm, atrial or ventricular septal defect, calcified aortic stenosis, and mitral annulus calcification [10,27,28].

Males and females differ with regard to cerebral ischemia. It has been shown that women with acute stroke, compared with men, had characteristic clinical features and natural history, with a worse prognosis. Emerging evidence suggests that therapeutic interventions based on the sex of patients may be promising, as some cell death pathways are sex-specific, and this may entail greater neuroprotection in adult ischemic brain injury [29,30]. However, to date, the specific analysis of clinical characteristics in the subgroup of women with cardioembolic cerebral infarction has been poorly characterized.

Preventive strategies and the in-hospital management of patients with cardioembolic stroke could benefit from the knowledge of gender differences. The present study adds evidence on clinical features and cerebrovascular risk factors for in-hospital mortality in a large cohort of women with cardioembolic cerebral infarct. The development of a predictive model of early death based on clinical data at admission and during hospital stay would allow the identification of at-risk patients who might especially benefit from very early treatment. To this end, we analyzed data from 4600 consecutive patients with acute stroke included in a prospective stroke registry over 24 years, and compared the frequency of demographic variables, risk factors, clinical events, neuroimaging data, and outcomes in women with cardioembolic stroke with those in male patients with cardioembolic ischemic stroke.

## 2. Materials and Methods

We performed a retrospective analysis of data on stroke patients prospectively collected in the hospital stroke registry of the Hospital Universitari Sagrat Cor, a 350-bed acute care university hospital in the city of Barcelona (Catalonia, Spain) serving an estimated population of 300,000 people [17]. For the present study, the data of all consecutive patients with cerebral infarction or primary cerebral hemorrhage admitted to the Department of Neurology, recorded over 24 years, were analyzed. This registry has been previously published and validated.

Data for each stroke patient admitted to the Department of Neurology of the hospital were collected using a 186-question protocolized questionnaire, including demographic data, cerebrovascular risk factors, personal history, findings of physical examinations, laboratory data, chest radiographs, 12-lead electrocardiogram, neuroimaging studies (non-contrast computed tomography (CT), CT angiography, angiography and/or magnetic resonance imaging), complications, and outcome on discharge. Other investigations, such as echocardiography, and lumbar puncture were performed at the discretion of the responsible neurologist.

For the classification of stroke subtypes, we used the criteria of the Catalan Society of Neurology and the Guidelines of the Cerebrovascular Study Group of the Spanish Society of Neurology [31]; both are similar to the classification of the National Institute of Neurological Disorders and Stroke [32] and have been used by our group in previous studies. The study obtained approval from the hospital’s Clinical Research Ethics Committee.

**Figure 1:**
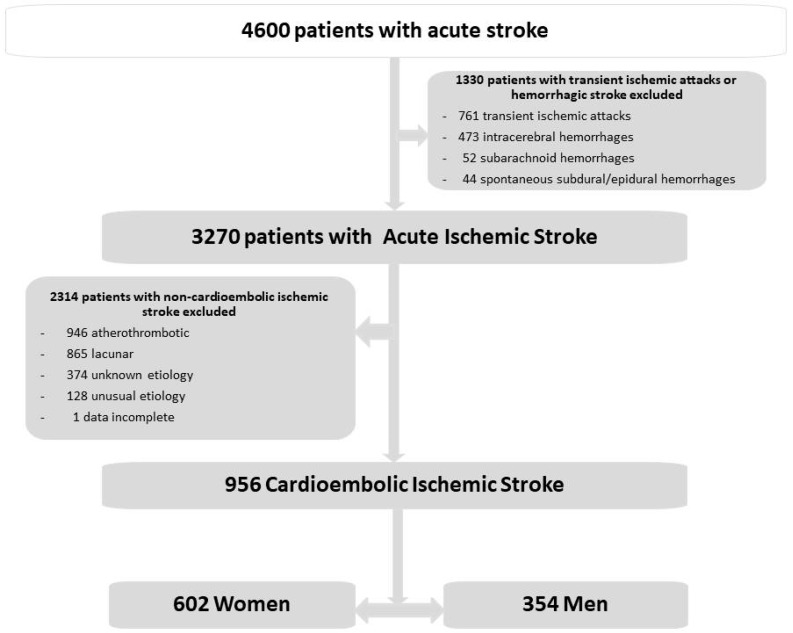
Flow chart of patients included in the study.

From an initial total of 4600 consecutive patients with acute stroke, 1330 patients were excluded, corresponding to 761 with transient ischemic attacks and 569 with hemorrhagic stroke (473 intracerebral hemorrhages, 52 subarachnoid hemorrhages, and 44 spontaneous subdural/epidural hemorrhages). There remained 3270 patients with acute ischemic stroke, of which 946 with atherothrombotic infarcts, 865 with lacunar infarcts, 128 with ischemic strokes of unusual etiology, 374 with infarcts of unknown etiology, and 1 patient with incomplete data were excluded. For the purposes of this study, and as shown in the flow diagram in Figure 1, only those patients diagnosed with cardioembolic ischemic infarction (n = 956) were selected: 602 women and 354 men.

The definition of cardioembolic cerebral infarction [32] coincides with the National Institute of Neurological Disorders and Stroke classification, which requires the presence of a cerebral infarction of medium (maximum lesion diameter of 1.5 to 3 cm) or large (>3 cm) size, the verification of cerebral cortex involvement on cerebral computed tomography (CT) and/or magnetic resonance imaging (MRI), usually sudden (minutes) or acute (hours) onset, onset during daily activities, maximal deficit at onset, focal neurological deficit >24 h, absence of clinical lacunar syndrome, and the identification of a commonly accepted cardiac source of embolus in the absence of confirmatory clinical (ipsilateral carotid bruit) or investigative (Doppler ultrasound, carotid angiography, or angio-MRI) findings of lesions (50% stenosis) in the ipsilateral supra-aortic trunks.

Patients were treated with the same clinical pathway after admission, in accordance with the recommendations of the Spanish Cerebrovascular Study Group of the Spanish Society of Neurology [32].

Medical complications (infectious, hemorrhagic, vascular, respiratory, cardiac, urinary, or sudden death) during the acute phase were assessed as well as the causes of early mortality. Vascular complications were deep venous thrombosis and peripheral arterial embolism; cardiac complications were heart failure, arrhythmia, and acute myocardial infarction; finally, pulmonary complications included superinfection, aspiration pneumonia, and pulmonary embolism. Mortality after stroke was assessed according to Silver et al. criteria [33]. The modified Rankin scale (mRs) for Neurologic disability was applied for evaluating the patient’s clinical status and degree of disability at discharge, and scored as spontaneous neurological improvement or good prognosis (mRS grades 0–2) and poor prognosis or in-hospital death [34]. 

### Statistical Analysis

Univariate analysis was performed for each variable in relation to vital status at discharge (alive or dead). Differences in the frequency of demographic characteristics, vascular risk factors, clinical events, and neuroimaging data, as well as outcome between female versus male patients were assessed. The chi-square (χ^2^) test (with Yates correction when necessary) or Fisher’s exact test was used for categorical variables, and Student’s *t* test and analysis of variance for the analysis of quantitative variables. Statistical significance was established at *p* < 0.05. 

Variables related to vital status at discharge in the univariate analysis plus age (used as a continuous variable with a constant odds ratio (OR) for each year) were subjected to multivariate analysis with a logistic regression procedure and forward stepwise selection when *p* was 0.10. In-hospital mortality, coded as alive = 0 and dead = 1, was the dependent variable.

The first predictive model was based on demographic and vascular risk factor variables. Clinical features were added in the second predictive model, and clinical and outcome variables were included in the third model. Statistically significant variables in the comparative analysis between female and male patients were also subjected to multivariate analysis. The maximum likelihood approach was used to estimate the logistic parameters weights [35,36]. IBM-SPSS Statistics Version 27 software for Windows, Armonk, NY, USA: IBM Corp., was used for statistical analysis [37,38].

## 3. Results

### 3.1. General Data

The study population included 956 consecutive patients diagnosed with cardioembolic acute ischemic stroke (602 women and 354 men). The frequency of different cardiac sources of emboli included atrial fibrillation (isolated or associated with structural cardiac disease) in 74.2% of patients, ischemic heart disease (acute myocardial infarction, left ventricular aneurysm, left ventricular ejection fraction < 40%, akinesia/dyskinesia ≥ 2 segments) in 20.8%, and rheumatic valvular disease in 16.4% of the patients. In women the mean age was 81.3 (SD 8.8) years versus 77.8 (SD 9.3) years in men. The frequency of very old patients (age ≥ 85 years) was significantly higher in women (37.7%) than in men (25.4%).

### 3.2. Differences between Cardioembolic Cerebral Infarct in Women and in Men

#### 3.2.1. Univariate Analysis

Table 1 shows the results of the univariate analysis of the differences between the groups of women and men with cardioembolic cerebral infarction. The results show a higher frequency of valvular heart disease, atrial fibrillation, heart failure, and limb weakness in the female group, whereas ischemic heart disease, chronic obstructive pulmonary disease, alcohol abuse, excessive smoking, and hyperlipidemia were significantly more frequent in the male group.

Internal capsule location was the most frequent lesion distribution of lesions in the female group versus vertebral artery vascular topography, more frequent in the male group. 

Early outcome showed greater focal neurological deterioration, together with a significantly less frequent absence of symptoms at hospital discharge and a higher percentage of severe limitation at hospital discharge in the women group. However, the in-hospital mortality rate remained statistically not significant.

#### 3.2.2. Multivariate Analysis

The multivariate analysis revealed the following as independent variables related to the female sex: obesity, valvular heart disease, hypertension, age, and internal capsule topography. On the other hand, the male sex showed as independent variables excessive smoking, chronic lung disease, ischemic heart disease, subacute onset, the location of the semioval center, and vertebral artery topography (Table 2).

**Figure 2:**
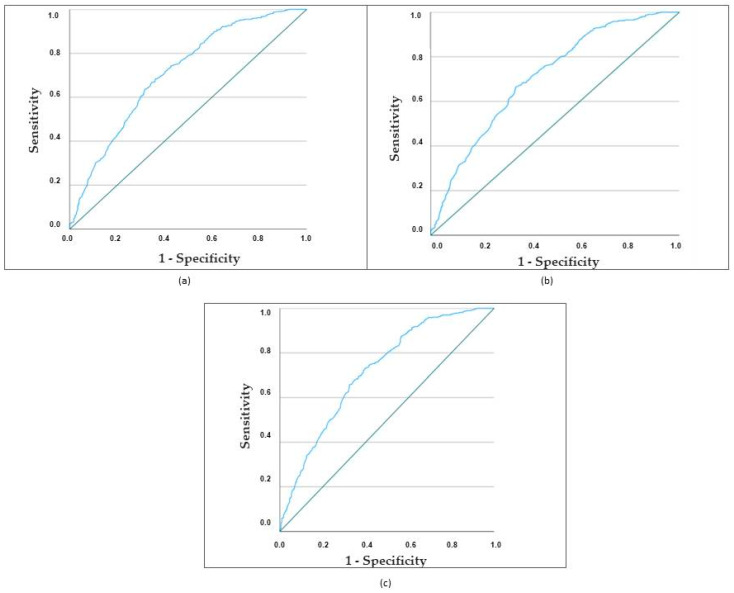
ROC curves of the three models. (**a**) ROC curve for the first regression model (demographic and risk factors). The AUC was 0.707. The sensitivity was 73%, specificity 58%, positive predictive value 74%, and negative predictive value 55%. Correctly classified in 67% of the cases. (**b**) ROC curve for the second regression model (demographic, risk factors, and clinical data). The AUC was 0.72. The sensitivity was 73%, specificity 59%, positive predictive value 75%, and negative predictive value 55%. Correctly classified in 67.2% of the cases. (**c**) ROC curve for the third regression model (demographic, risk factors, clinical, brain topography, and early outcome). The AUC was 0.719. The sensitivity was 72%, specificity 61%, positive predictive value 76%, and negative predictive value 56%. Correctly classified in 67.8% of the cases.

According to these three models, cases of cardioembolic stroke in women were correctly classified in 67.0%, 67.2%, and 67.8% of cases, respectively. Figure 2 illustrates the receiver operating characteristic (ROC) curves of the three models, showing that the three ROC curves of the models present a similar AUC (0.707, 0.72, and 0.719). The differences between the three models are not significant: Model 1 versus Model 2: *p* = 0.478; Model 1 versus Model 3: *p* = 0.125; and Model 2 versus Model 3: *p* = 0.177.

### 3.3. Predictors of In-Hospital Mortality in Ischemic Cardioembolic Stroke in Women

#### 3.3.1. Univariate Analysis

The overall in-hospital mortality of the 602 women with ischemic cardioembolic stroke was 23.1% (n = 139). The causes of death were non-neurological in 54% of cases: pneumonia (15%), cardiac disease (14%), pulmonary thromboembolism (14%), sepsis (7%), sudden death (3%), and mesenteric artery embolism (1%); they were neurological in 39.5% of cases, corresponding to cerebral herniation (25%), recurrent cerebral ischemia (8%), and cerebral hemorrhage (6.5%). The cause was unknown in 6.5% of cases.

In the univariate analysis, the variables significantly associated with outcome are shown in Table 3.

#### 3.3.2. Multivariate Analysis

After the multivariate analysis, age, previous ischemic stroke, early seizures, altered consciousness, limb weakness, and medical complications (neurological, respiratory, gastrointestinal, kidney, cardiac, and vascular) appeared to be independent variables to in-hospital death (Table 4).

**Figure 3:**
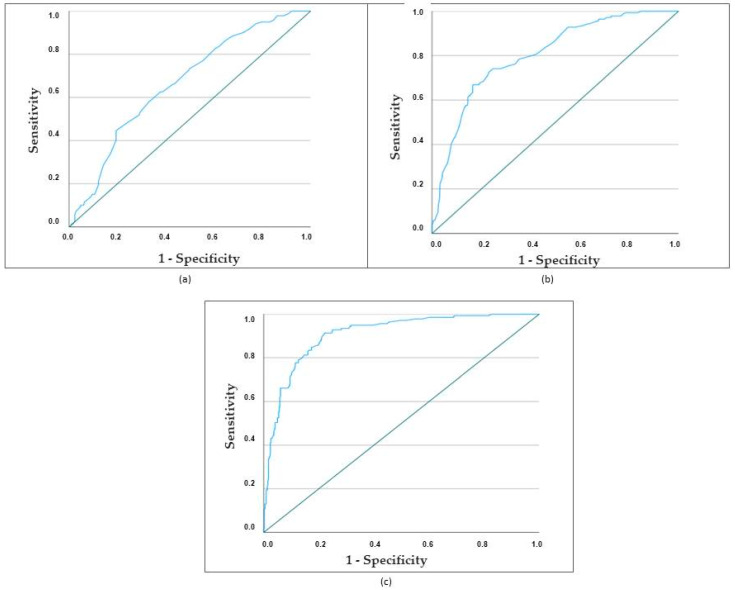
ROC curves of the three models. (**a**) ROC curve for the first regression model based on demographic and risk factors. The area under the curve (AUC) was 0.666. The sensitivity was 66%, specificity 57%, positive predictive value 32%. and negative predictive value 85%. Correctly classified in 59.1% of the cases. (**b**) ROC curve for the second regression model based on demographic, risk factors, and clinical features. The area under the curve (AUC) was 0.803. The sensitivity was 68%, specificity 79%, positive predictive value 50%, and negative predictive value 89%. Correctly classified in 76.7% of the cases. (**c**) ROC curve for the third regression model based on demographic, risk factors, clinical features, location, vascular topography, and complications. The area under the curve (AUC) was 0.908. The sensitivity was 81%, specificity 79%, positive predictive value 57%, and negative predictive value 57%. It was correctly classified in 82% of the cases.

According to these three models, the mortality of cardioembolic stroke in women was correctly classified in 59.1%, 67.8%, and 82% of cases, respectively. Figure 3 illustrates the receiver operating characteristic (ROC) curves of the three models, and the AUC of the three models was 0.666, 0.803, and 0.908. This difference is clinically relevant and statistically significant: Model 1 versus Model 2: *p* = 0.000; Model 1 versus Model 3: *p* = 0.000; and Model 2 versus Model 3: *p* = 0.000.

The area under the curve (AUC) of the third model based on demographics, risk factors, clinical features, location, vascular topography, and complications was 0.908. The sensitivity was 86%, specificity 81%, positive predictive value (PPV) 57%, and negative predictive value (NPV) 57%.

## 4. Discussion

### 4.1. General Considerations

The main finding of our study was that ischemic cardioembolic stroke (CS) has a sex-related modifying effect on clinical and early outcome: women with CS were markedly older and differed from men in the distribution of cerebrovascular risk factors, clinical data, and early stroke severity.

Cardioembolic cerebral infarction is a devastating stroke subtype, with the highest in-hospital mortality during the acute phase of stroke and with high rates of long-term disability [39,40]. Increasingly, the study of the different variables according to sex is becoming important. However, and despite updated National Institutes of Health (NIH) policies since 2016 requiring research studies to consider sex as a biological variable [41], data remain limited.

In our experience, analyzing data collected in our acute stroke registry for 24 consecutive years, the comparative analysis reflects markedly different demographics, risk factors, and clinical features between women and men, with worse early prognosis in women. 

The analysis of demographic data related to sex in CS is limited. Our results reveal that CS in women develops mainly at advanced ages (mean age 81 years) and that, in turn, CS in the oldest group (85 years or older) is more frequent in women (37.7%) than in men (25.4%). This implies an increase in the number of female stroke victims in Western societies, due to the greater life expectancy of women and the higher incidence of stroke at older ages [42,43,44].

### 4.2. Biomarkers and Molecular Biological Features

Genetic, epigenetic, and hormonal processes of biological sex influence physiology and disease. Gallego-Fabrega et al. [45] point out the importance of finding specific biological mechanisms involved in stroke risk in women and measured epigenetic age acceleration (EAA) based on DNA methylation (DNAm) age predictors, and suggest that biological age acceleration is lower in women with ischemic stroke compared to men. Women have decreased values of epigenetic age acceleration, indicating that their biological age is lower than expected at the time of ischemic stroke onset, contrary to the higher epigenetic age acceleration in men, who are biologically older than their chronological age.

Women are on average younger than their chronological age, whereas men show positive age acceleration.

Another widespread hypothesis about the sexual dichotomy in the occurrence of stroke is that steroid hormones, in particular estrogens, have tissue antioxidant properties that could contribute to neuroprotection during ischemic episodes. Thus, the risk of stroke is lower in premenopausal women than in men of the same age [46,47]. In addition, estrogens have a beneficial effect on serum lipids and coagulation profile and in the prevention of coronary heart disease. After menopause, there is an increased incidence of stroke in women due to estrogen-induced alterations in lipid metabolism, so that lower estrogen levels reduce the anti-inflammatory and neuroprotective effects of the hormone [48,49,50]. Menopause also accelerates epigenetic aging.

Gender-specific differences disappear in the 65–84 age group and reappear in the oldest group (≥85 years), being greater in women globally [51,52,53]. Reduced estrogen neuroprotection may aggravate brain damage and consequently increase morbidity and poor prognosis in women with CS. Regarding the primary endpoints of the study, differences in early outcomes between women and men support a sex-related modifying effect on the prognosis of CS in women.

The sex dichotomy of age at stroke onset could be influenced by a combination of intrinsic biological processes and lifestyle or sociocultural factors (body mass index, education, diet, and income).

### 4.3. Cardioembolic Cerebral Infarct in Women and in Men: Differences and Outcome

Risk factors reveal similarities, but also important differences between CS in women and men. Both coincide in atrial fibrillation as the most important risk factor for CS stroke [54,55,56]. From this point on, in our study, the variables with independent predictive value in female patients were obesity, valvular heart disease, and hypertension. This is consistent with the results of Andersen et al., who also reported a higher frequency of hypertension and obesity, particularly abdominal obesity, in a nationwide study in Denmark [57].

Ischemic heart disease, chronic obstructive pulmonary disease, and heavy smoking continue to have a lower incidence in women and, in turn, they remain the leading preventable causes of CS in men [52,53,54,55].

It is unclear whether the influence of sex on the length of hospital stay or outcome reflects the impact of cultural difference [58]. However, regarding the controversy on whether women with CS have worse early prognosis than men, in our study, women had a higher severity of stroke. Older age and internal capsular topography, more frequent in women, could partly explain these data, due to the disruption of the pyramidal tract and the possible compression of adjacent thalamocortical sensory projections. Capsular involvement in non-lacunar ischemic stroke can be severely disabling. This phenomenon could be explained by the higher density of corticospinal tracts in these regions [59,60,61]. In addition, the involvement of the internal capsule could be an expression of larger brain lesions in women, as proposed by Eriksson et al. [62].

Another relevant finding in our study was the dominant relevance of medical complications developed during hospital care in predicting in-hospital mortality in female acute CS victims. Neurological, respiratory, gastrointestinal, renal, cardiac, and peripheral vascular complications were independent predictors related to early mortality in women with CS. The verification of these variables is easily obtained during a bedside examination.

The presence of infections of any type is a cause of acute neurological deterioration, increasing both mortality and morbidity in patients with acute stroke [63,64]. Stroke-associated infection, particularly pneumonia, has been shown to be independently associated with poor functional outcome after ischemic stroke. Urinary episodes, such as urinary tract infection, incontinence or urinary retention, are the most frequent complications in stroke patients and may be the origin of bloodstream infection [63,64]. Deep venous thrombosis is the most frequent vascular complication and can lead to pulmonary thromboembolism, which is a frequent cause of death during the acute phase of stroke. The presence of any of these complications should be diagnosed and appropriate pharmacological treatment instituted; these are circumstances that lengthen hospital stay [65].

Other clinical predictors related to the severity of CE stroke, such as limb weakness, altered consciousness, or advanced age, are consistent with data reported in other general studies of acute stroke [66,67,68,69,70,71,72,73,74].

However, the results of the present study should be interpreted taking into account some limitations. First, there could be a selection bias due to the single-center design. In this regard, future large multicenter studies should be recommended. Second, the mRS was the main test used to assess disability. It is an easy test to homogeneously quantify the functional limitation of the patients; however, other functional tests could show a more accurate functional disability. Another limitation of this study would be not to have analyzed the prognostic value of sex in medium- or long-term CE stroke. In future studies, these aspects would be interesting lines of research.

It should be mentioned, however, that our study also has strengths, since it is a large and well-characterized cohort, with complete baseline clinical data and early results, and despite being retrospective, it was performed with minimal losses during follow-up. The evaluation of the methods used is more objective than not.

Another promising line of research would be the use of biomarkers to better understand prognosis and early outcome. Cerebral atrophy is a new emerging feature of cerebrovascular diseases, and this gray matter atrophy is usually progressive and documented mainly in patients with acute cerebral small vessel disease [75,76,77]. The sex-related modifying effect on the clinical and early prognosis of acute SC in relation to cerebral atrophy should be further investigated in future research.

## 5. Conclusions

Our findings indicate that women with cardioembolic stroke differ from men in several respects. Whereas heavy smoking, chronic obstructive pulmonary disease, and ischemic heart disease occurred more frequently in male patients with CS, predictors of CS in women were age, obesity, valvular heart disease, and hypertension. Consequently, measures to correct obesity and control hypertension and heart disease are particularly important therapeutic strategies to prevent acute CS in women.

Another point to highlight is that women have worse early outcomes than men, and that medical complications (neurological, vascular, urinary, and infectious) are relevant factors influencing in-hospital mortality after acute CS. Therefore, the early recognition of complications is essential, as the vast majority of patients who suffer medical complications have a poor short-term prognosis.

In summary, women with acute cardioembolic ischemic infarction are a subgroup of stroke patients with an unfavorable early outcome associated with older age, impaired consciousness, and medical complications that develop during hospitalization.

In cardioembolic infarction, there are important differences between males and females in terms of symptoms, the course of the disease, and therapeutic response due to molecular, genetic, and epigenetic mechanisms related to biological sex.

Efforts to determine sex specificities in stroke incidence and prognosis, as well as the differential effects of other risk factors, are important to individualize stroke prevention and treatment. Emerging lines of research in female patients with CS are blood biomarkers, the relevance of brain atrophy, and medium- and long-term follow-up.

A better understanding of the impact of sex and gender on acute cardioembolic stroke will improve outcomes for all individuals.

## Figures and Tables

**Table 1:** Cerebrovascular risk factors, clinical findings, neuroimaging, and outcome in women and in men with acute ischemic cardioembolic stroke.

Variables	Men (n = 354)	Women (n = 602)	*p* Value
Cerebrovascular risk factors			
Hypertension	173 (48.9)	340 (56.5)	0.023
Diabetes mellitus	71 (20.1)	106 (17.6)	0.347
Valvular heart disease	40 (11.3)	117 (19.4)	0.001
Ischemic heart disease	108 (30.5)	91 (15.1)	0.000
Atrial fibrillation	248 (70.1)	462 (76.7)	0.022
Heart failure	27 (7.6)	73 (12.1)	0.028
Previous ischemic stroke	74 (20.9)	100 (16.6)	0.097
Chronic obstructive pulmonary disease	50 (14.1)	34 (5.6)	0.000
Peripheral vascular disease	31 (8.8)	37 (6.1)	0.129
Obesity	6 (1.7)	22 (3.7)	0.083
Alcohol abuse (>80 gr/day)	10 (2.8)	0 (0.0)	0.000
Anticoagulants	36 (10.2)	69 (11.5)	0.537
Heavy smoking (>20 cigarettes/day)	38 (10.7)	4 (0.7)	0.000
Hyperlipidemia	59 (16.7)	71 (11.8)	0.034
Clinical findings			
Sudden onset	234 (66.1)	390 (64.8)	0.679
Acute onset (hours)	68 (19.2)	133 (22.1)	0.291
Subacute onset (>24 h)	24 (6.8)	27 (4.5)	0.127
Headache	32 (9.0)	41 (6.8)	0.210
Vertigo	12 (3.4)	15 (2.5)	0.418
Seizures	5 (1.4)	12 (2.0)	0.512
Nausea, vomiting	29 (8.2)	33 (5.5)	0.100
Altered consciousness	92 (26.0)	172 (28.6)	0.388
Limb weakness	266 (75.1)	495 (82.2)	0.009
Sensory deficit	123 (34.7)	223 (37.0)	0.475
Hemianopia	74 (20.9)	128 (21.3)	0.896
Speech disorders (dysarthria, aphasia)	206 (58.2)	383 (63.6)	0.096
Extrapyramidal symptoms	7 (2.0)	10 (1.7)	0.721
Neuroimaging finding topography			
Frontal lobe	74 (20.9)	139 (23.1)	0.433
Parietal lobe	116 (32.8)	235 (39.0)	0.052
Temporal lobe	139 (39.3)	246 (40.9)	0.627
Occipital lobe	43 (12.1)	70 (11.6)	0.810
Internal capsule	36 (10.2)	95 (15.8)	0.015
Thalamus	12 (3.4)	23 (3.8)	0.732
Basal ganglia	44 (12.4)	96 (15.9)	0.137
Centrum semiovale	7 (2.0)	5 (0.8)	0.216
Anterior cerebral artery	20 (5.6)	25 (4.2)	0.291
Middle cerebral artery	219 (61.9)	401 (66.6)	0.138
Posterior cerebral artery	30 (8.5)	47 (7.8)	0.714
Basilar artery	10 (2.8)	13 (2.2)	0.517
Vertebral artery	13 (3.7)	6 (1.0)	0.004
Outcome			
Neurological complications	44 (12.4)	82 (13.6)	0.599
Respiratory complications	51 (14.4)	87 (14.5)	0.985
Gastrointestinal complications	11 (3.1)	17 (2.8)	0.802
Urinary tract complications	33 (9.3)	53 (8.8)	0.787
Cardiac complications	33 (9.3)	53 (8.8)	0.787
Vascular complications	11 (3.1)	16 (2.7)	0.685
Hemorrhagic complications	11 (3.1)	16 (2.7)	0.685
Infectious complications *	57 (16.1)	115 (19.1)	0.243
Symptom free at discharge	57 (16.1)	67 (11.1)	0.027
Poor outcome (mRS grades 4–5)	43 (12.1)	105 (17.4)	0.029
In-hospital death	79 (22.3)	139 (23.1)	0.783
Length of hospital stay—days, mean (SD)	17.89 (14.88)	18.21 (14.38)	0.709

Values are mean ± SD or n (%). * Other than urinary tract infection.

**Table 2:** Results of multivariate analysis: independent variables associated with acute cardioembolic ischemic stroke in women.

Regression Models	Coefficient (β)	Standard Error	Odds Ratio (95% CI)	*p* Value
First model: demographics and risk factors				
Age	0.044	0.008	1.05 (1.03–1.06)	0.000
Hypertension	0.372	0.146	1.45 (1.09–1.93)	0.011
Valvular heart disease	0.790	0.216	2.20 (1.44–3.36)	0.000
Ischemic heart disease	−0.764	0.174	0.47 (0.33–0.66)	0.000
Chronic obstructive pulmonary disease	−1.004	0.254	0.37 (0.22–0.60)	0.000
Obesity	1.213	0.528	3.36 (1.19–9.47)	0.022
Heavy smoking (>20 cigarettes/day)	−2.477	0.544	0.08 (0.03–0.24)	0.000
Second model: demographics, risk factors, and clinical features				
Age	0.045	0.008	1.05 (1.03–1.06)	0.000
Hypertension	0.369	0.146	1.45 (1.09–1.93)	0.012
Valvular heart disease	0.771	0.217	2.16 (1.41–3.31)	0.000
Ischemic heart disease	−0.773	0.174	0.46 (0.33–0.65)	0.000
Chronic obstructive pulmonary disease	−1.019	0.254	0.36 (0.22–0.60)	0.000
Obesity	1.184	0.530	3.27 (1.16–9.23)	0.025
Heavy smoking (>20 cigarettes/day)	−2.506	0.544	0.08 (0.03–0.24)	0.000
Subacute onset	−0.635	0.302	0.53 (0.29–0.96)	0.036
Third model: demographics, risk factors, clinical features, location, vascular topography, and complications				
Age	0.045	0.008	1.05 (1.03–1.06)	0.000
Hypertension	0.409	0.148	1.51 (1.13–2.01)	0.006
Valvular heart disease	0.734	0.218	2.08 (1.36–3.20)	0.001
Ischemic heart disease	−0.823	0.176	0.44 (0.31–0.62)	0.000
Chronic obstructive pulmonary disease	−1.004	0.256	0.37 (0.22–0.61)	0.000
Obesity	1.164	0.533	3.20 (1.13–9.10)	0.029
Heavy smoking (>20 cigarettes/day)	−2.540	0.549	0.08 (0.03–0.23)	0.000
Subacute onset	−0.630	0.306	0.53 (0.29–0.97)	0.039
Internal capsule	0.502	0.229	1.65 (1.06–2.59)	0.028
Centrum semiovale	−1.226	0.610	0.29 (0.09–0.97)	0.044
Vertebral artery	−1.104	0.543	0.33 (0.11–0.96)	0.042

Model 1: Hosmer–Lemeshow goodness-of-fit test 0.586, correctly classified 67.0% of cases; Model 2: Hosmer–Lemeshow goodness-of-fit test 0.362, correctly classified 67.2% of cases; Model 3: Hosmer–Lemeshow goodness-of-fit test 0.786, correctly classified 67.8% of cases.

**Table 3:** Results of a univariate analysis in 602 female patients with cardioembolic stroke.

Variables	Alive (n = 463)	Dead (n = 139)	*p*
Demographics			
Age—years, mean (SD)	80.22 (9.21)	84.71 (6.27)	0.000
≥85 years old—no. (%)	158 (34.1%)	69 (49.6%)	0.001
Cerebrovascular risk factors			
Hypertension	270 (58.3)	70 (50.4)	0.097
Diabetes mellitus	84 (18.1)	22 (15.8)	0.530
Valvular heart disease	95 (20.5)	22 (15.8)	0.220
Ischemic heart disease	71 (15.3)	20 (14.4)	0.785
Atrial fibrillation	350 (75.6)	112 (80.6)	0.223
Heart failure	52 (11.2)	21 (15.1)	0.219
Previous ischemic stroke	68 (14.7)	32 (23.0)	0.021
Chronic obstructive pulmonary disease	22 (4.8)	12 (8.6)	0.082
Peripheral vascular disease	26 (5.6)	11 (7.9)	0.322
Obesity	18 (3.9)	4 (2.9)	0.578
Heavy smoking (>20 cigarettes/day)	3 (0.6)	1 (0.7)	1.000
Hyperlipidemia	60 (13.0)	11 (7.9)	0.106
Clinical features			
Sudden onset	296 (63.9)	94 (67.6)	0.424
Acute onset (hours)	107 (23.1)	26 (18.7)	0.272
Headache	34 (7.3)	7 (5.0)	0.344
Vertigo	14 (3.0)	1 (0.7)	0.223
Seizures	5 (1.1)	7 (5.0)	0.010
Nausea, vomiting	23 (5.0)	10 (7.2)	0.312
Altered consciousness	83 (17.9)	89 (64.0)	0.000
Limb weakness	366 (79.0)	129 (92.8)	0.000
Sensory deficits	163 (35.2)	60 (43.2)	0.088
Hemianopia	95 (20.5)	33 (23.7)	0.415
Speech disorders (aphasia, dysarthria)	305 (65.9)	78 (56.1)	0.036
Extrapyramidal symptoms	10 (2.2)	0 (0.0)	0.171
Brain topography			
Frontal lobe	101 (21.8)	38 (27.3)	0.175
Parietal lobe	161 (34.8)	74 (53.2)	0.000
Temporal lobe	174 (37.6)	72 (51.8)	0.003
Occipital lobe	50 (10.8)	20 (14.4)	0.247
Internal capsule	69 (14.9)	26 (18.7)	0.281
Thalamus	20 (4.3)	3 (2.2)	0.244
Basal ganglia	60 (13.0)	36 (25.9)	0.000
Vascular topography			
Anterior cerebral artery	18 (3.9)	7 (5.0)	0.552
Middle cerebral artery	298 (64.4)	103 (74.1)	0.033
Posterior cerebral artery	41 (8.9)	6 (4.3)	0.080
Basilar artery	8 (1.7)	5 (3.6)	0.319
Vertebral artery	2 (0.4)	4 (2.9)	0.040
Early outcome			
Neurological complications	28 (6.0)	54 (38.8)	0.000
Respiratory complications	35 (7.6)	52 (37.4)	0.000
Gastrointestinal complications	6 (1.3)	11 (7.9)	0.000
Renal complications	5 (1.1)	6 (4.3)	0.033
Urinary tract complications	42 (9.1)	11 (7.9)	0.673
Cardiac complications	23 (5.0)	30 (21.6)	0.000
Vascular complications	9 (1.9)	7 (5.0)	0.092
Hemorrhagic complications	9 (1.9)	7 (5.0)	0.092
Infectious complications *	66 (14.3)	49 (35.3)	0.000

* Other than urinary tract infection.

**Table 4:** Independent risk factors for in-hospital mortality in 602 women with cardioembolic ischemic cerebral infarction.

Regression Model	Coefficient (β)	Standard Error	Odds Ratio (95% Confidence Interval)	*p* Value
Model based on demographics and risk factors				
Age	0.073	0.014	1.08 (1.05–1.08)	0.000
Previous ischemic stroke	0.560	0.248	1.75 (1.08–2.85)	0.024
Model based on demographics, risk factors, and clinical features				
Age	0.064	0.016	1.07 (1.03–1.10)	0.000
Early seizures	1.818	0.704	6.16 (1.55–24.47)	0.010
Altered consciousness	1.952	0.222	7.05 (4.56–10.90)	0.000
Limb weakness	1.098	0.376	3.00 (1.44–6.26)	0.003
Model based on demographics, risk factors, clinical features, location, vascular topography, and complications				
Age	0.060	0.019	1.06 (1.02–1.10)	0.001
Altered consciousness	1.946	0.272	7.00 (4.11–11.92)	0.000
Limb weakness	0.931	0.428	2.54 (1.10–5.87)	0.030
Neurological complications	2.266	0.327	9.64 (5.08–18.30)	0.000
Respiratory complications	1.879	0.316	6.55 (3.53–12.16)	0.000
Gastrointestinal complications	1.415	0.673	4.12 (1.10–15.39)	0.035
Renal dysfunction	2.020	0.907	7.54 (1.27–44.62)	0.026
Cardiac complications	1.785	0.403	5.96 (2.71–13.12)	0.000
Vascular complications	1.478	0.652	4.39 (1.22–15.73)	0.023

Model 1: Hosmer–Lemeshow goodness-of-fit test 0.314, correctly classified 59.1% of cases; Model 2: Hosmer–Lemeshow goodness-of-fit test 0.502, correctly classified 76.7% of cases; Model 3: Hosmer–Lemeshow goodness-of-fit test 0.766, correctly classified 82.0% of cases.

## Data Availability

Data are contained within the article.
